# Efficacy of a Native Microbial Starter in Promoting Table Olive Fermentation: An Industrial-Scale Trial at Controlled and Ambient Temperature

**DOI:** 10.3390/foods14132159

**Published:** 2025-06-20

**Authors:** Marco Campus, Francesco Corrias, Alberto Angioni, Nicola Arru, Piergiorgio Sedda, Margherita Addis, Myriam Fiori, Antonio Paba, Luigi Chessa, Roberta Comunian

**Affiliations:** 1 AGRIS—Agricultural Research Agency of Sardinia, 07100 Sassari, Italy; psedda@agrisricerca.it (P.S.); maddis@agrisricerca.it (M.A.); mfiori@agrisricerca.it (M.F.); apaba@agrisricerca.it (A.P.); lchessa@agrisricerca.it (L.C.); rcomunian@agrisricerca.it (R.C.); 2 Food Toxicology Unit, Department of Life and Environmental Science, Campus of Monserrato, University of Cagliari, 09042 Cagliari, Italy; aangioni@unica.it (A.A.); nicola.arru@unica.it (N.A.)

**Keywords:** table olive, microbial starter, fermentation, food processing

## Abstract

This study evaluated a multi-strain starter culture’s impact on the industrial-scale fermentation of “Tonda di Cagliari” table olives, comparing processes at ambient versus controlled (23–25 °C) temperatures. Controlled fermentation accelerated acidification, yielding lower pH levels, higher lactic acid bacteria (LAB) counts, and better control over Enterobacteriaceae. Starter inoculation ensured the attainment of safe pH levels (<4.2) even at ambient temperature, while uninoculated samples did not reach safe pH levels under those conditions (>4.5 in non-inoculated samples). Regardless of processing temperature, starter-inoculated olives consistently yielded higher final concentrations of hydroxytyrosol (719.2 and 762.9 mg/kg inoculated, 480.7 and 326 mg/kg non-inoculated). Total phenolic content in olives remained higher throughout the fermentation process at the controlled temperature (3138 and 2112 mg/kg ambient temperature, 3458 and 3622 mg/kg controlled temperature). Olives maintained at controlled (higher) temperatures exhibited lower final moisture content and significantly reduced lipid content. While texture profiles were primarily affected by temperature, sensory acceptability was significantly influenced by both the starter inoculation and the fermentation temperature. These findings indicate that using microbial starters can potentially lower energy costs associated with heating processing rooms, particularly during colder seasons, while still ensuring food safety and enhancing nutraceutical value. Although the faster fermentation rate at controlled temperature did not substantially shorten overall marketing time, the starter eliminates the necessity for heating facilities to achieve a food-safe pH within a reasonable timeframe.

## 1. Introduction

The production of table olives requires meticulous planning and management of the fermentation process to ensure both the quality and shelf-life of the final product [1]. When properly conducted, fermentation ensures the safety of the olives for consumption, enhances their flavor, and promotes the development of a desirable texture. During fermentation, microorganisms convert simple carbohydrates into lactic acid, which diffuses from the olive into the brine, lowering the pH of the fermentation mixture and preventing spoilage. Olive fermentation involves diverse microbial populations, with lactic acid bacteria (LAB) and yeasts being the dominant species. Other microorganisms, such as *Enterobacteriaceae*, *Clostridium*, *Pseudomonas*, *Staphylococcus*, occasional molds and other species originating from salt and processing environment, may also be present. The characteristics of the final product, including flavor, texture, and safety, are largely determined by these microorganisms and their metabolic activities [2]. Traditionally, table olive fermentation occurs spontaneously, driven by the olive’s natural microbiota, without the addition of starter cultures. In general, LAB and/or yeasts are the main agents of brine acidification, producing lactic acid from fermentable substrates. This process lowers the pH, enhances microbiological stability, and extends the product’s shelf life. Yeasts, conversely, primarily contribute to the aroma and flavor profile by generating desirable metabolites and volatile compounds. They also support LAB growth and facilitate the degradation of phenolic compounds. Traditional fermentation methods, such as the Greek and Spanish processes, rely on spontaneous fermentation at ambient temperature. These methods are slow, often requiring months to complete, and are associated with a high risk of proliferation of undesired microorganisms, primarily due to an insufficient or inconsistent presence of lactic acid bacteria (LAB) on the olives’ surface or in the brine, which can result in incomplete or irregular fermentations. Alternatively, fermentation can be initiated by introducing a portion of a previously successful fermentation batch into a new one (backslopping), or by adding specific starter cultures. The microorganisms used in the production of fermented foods belong to various taxonomic groups, predominantly lactic acid bacteria (LAB), including both homofermentative and heterofermentative types, as well as micrococci, propionibacteria, yeasts, and molds [3]. Selecting appropriate microorganisms for table olive fermentation requires careful consideration of several factors. The optimal species often depends on the olive cultivar, the processing method, and the geographical origin of the raw material [4]. Additional criteria for selecting starter strains include the microorganism’s growth rate, its capacity to withstand challenging brine conditions (such as low temperatures, high pH, and elevated NaCl concentrations), its ability to adhere to the olive cuticle, and its survival after freeze-drying [5]. Other important factors are resistance to organic acids and polyphenols, efficiency in rapidly metabolizing fermentable substrates and oleuropein, the ability to produce desirable aromas, bacteriocins synthesis, and the capacity to colonize the brine while inhibiting the growth of undesirable microorganisms (e.g., *Enterobacteriaceae*, *Clostridium*, *Pseudomonas*, *Staphylococcus*, and *Listeria*) [3]. Moreover, selected microorganisms should be recognized by qualified experts as safe for their intended use (Generally Recognized As Safe, GRAS, by Food and Drug Administration—FDA), or possess Qualified Presumption of Safety (QPS) status from European Food Safety Authority (EFSA). The scientific literature on the biodiversity of table olive microflora provides comprehensive lists of bacterial, yeast, and mold species [6,7,8,9]. To date, starter cultures for table olive, primarily developed for Spanish-style processing, typically consist of one or two strains from facultative heterofermentative mesophilic lactobacilli. The most prominent among these are *Lactiplantibacillus plantarum* and *Lactiplantibacillus pentosus*, with *Lactiplantibacillus paraplantarum*, *Lacticaseibacillus casei*, *Lacticaseibacillus brevis*, *Leuconostoc mesenteroides*, and *Pediococcus acidilactici* being used less frequently, and Enterococci only rarely. Occasionally, bacteria are combined with yeasts such as *Debaryomyces* spp., *Saccharomyces* spp., *Candida* spp., *Pichia* spp., and *Rhodotorula* spp., to promote and enhance their growth and fermentation activity [2,4,5]. The introduction of microbial starters, based on LAB used alone or in combination with yeasts, represents an emerging, safer, and more cost-effective alternative [10,11,12]. This approach optimizes the fermentation process, significantly improving the safety and the overall quality of the product. The use of microbial starters offers several advantages, including accelerated acidification with a faster and more consistent fermentation process [2,13,14], enhanced production of bioactive compounds such as antimicrobial peptides, organic acids, and bacteriocins, which inhibit spoilage microorganisms and further enhance the safety of table olives [3]. Moreover, LAB ensures greater uniformity in taste and texture, improves sensory properties, and reduces the risk of off-flavors or sensory defects [15]. The accelerated fermentation process depends on the microbial strains used, temperature conditions, and processing techniques [16], and it significantly enhances production efficiency and consistency across production cycles, enabling producers to meet market demands more rapidly and effectively [6]. This approach provides a cost-effective solution for maintaining optimal product quality, which is a critical factor for large-scale manufacturers. Furthermore, it aligns with the growing consumer demand for reduced use of synthetic chemical preservatives, offering a healthier and more environmentally sustainable food option [12]. Much of the current research has been conducted under laboratory conditions; therefore, there remains a need for scaling up studies to validate the efficiency of microbial starters in industrial settings [17]. In this paper, the results of an industrial-scale study examining the efficacy of an autochthonous multi-strain microbial starter, consisting of a mix of *Lactiplantibacillus pentosus* strains, previously characterized [9] and tested under laboratory conditions, are presented [18]. This study compares the starter-driven process with natural fermentation by monitoring relevant chemical and physical parameters, including phenolic compounds, lipids, tocopherols, and chlorides. Additionally, the impact of the processing technology on overall quality was assessed through consumer testing and instrumental measurement of texture parameters.

## 2. Materials and Methods

### 2.1. Starting Material and Processing Conditions

Olives of the “Tonda di Cagliari” variety coming from an irrigated olive grove located in Sardinia (Italy) were harvested before the veraison at the yellow-green stage, (Jaén maturation index 1), washed in tap water under stirring, then calibrated. After dripping the excess of water, olives were placed in sanitized plastic barrels with a capacity of 220 L (130 kg of olives and 90 L of 8% NaCl *w*/*v* brine, barrels *n*. = 12), the salt concentration was kept constant throughout the process by monitoring and periodical refill of the salt in the barrels. Six barrels were inoculated with a mix of *Lactiplantibacillus pentosus* strains as starter, at the concentration of 5 Log CFU/mL, selected from spontaneously fermented olives and previously characterized for their technological features [18] (series I), whereas six non inoculated barrels were used as control (series C). Three barrels of series I and three of series C were placed in a heated room set at 25 °C, at the Agris experimental plant (VI: inoculated; VC: uninoculated). The remaining three barrels of each experiment (ZI: inoculated; ZC: control) were kept at ambient temperature (20 °C ± 8 °C) in an industrial production farm. The experimentation period lasted 160 days, during which the fermentation process was monitored, and olive samples were taken for compositional analyses at selected times (0, 7, 14, 30, 60, 90, and 160 days).

### 2.2. Reagents and Standards

Methanol, chloroform, acetonitrile, heptane, hexane, and TerButylMethylEther (TBME) were all LC/MS-grade solvents purchased from Carlo Erba (Milan, Italy). H3PO4 and Folin–Ciocalteu reagent were all from Carlo Erba (Milan, Italy). Sodium carbonate, potassium chloride, K_2_CrO_4_, AgNO_3_ 0.1 N, and α-tocopherol were purchased from Sigma Aldrich (Milan, Italy). Double-deionized water with a conductivity of less than 18.2 MΩ was obtained with a Milli-Q system (Millipore, Bedford, MA, USA).

### 2.3. Physicochemical Analysis on Fermentation Brines

The analysis of pH was carried out using a pH-meter (420 A, Orion, Boston, MA, USA). Sodium chloride content, in brines, was determined by potentiometric titration with AgNO_3_ 0.1 N by using an automatic titrator (Mettler-Toledo DL55, Mettler-Toledo GmbH, Schwerzenbach, Switzerland).

### 2.4. Olives Composition

#### 2.4.1. Sample Pre-Treatment

Table olive samples, 130 g of olives and 90 mL of fermentation brine, were taken from each fermentation barrel at selected times. Olives were drained of the brine, rinsed, pitted, and finely ground with a knife and successively homogenized in a cold bath until a smooth paste was obtained. The analysis for the determination of the phenolic fraction was performed immediately to avoid loss of compounds by oxidation. The remaining samples were placed in a 50 mL falcon tube and frozen.

#### 2.4.2. Moisture

Homogenized samples (2 g) were weighed and dried at 105 °C in a thermostatic oven (9000 series-RS232, Isco, Milan, Italy) until a constant weight was achieved (∼24 h). After that, samples were stored in a desiccator at ambient temperature and weighed moisture was expressed as g/100 g fresh weight (FW).

#### 2.4.3. Total Lipids

Total lipids were determined according to a modified Folch method. Briefly, 200 mg of homogenized sample was weighed in a 15 mL falcon tube plus 1.5 mL of methanol and 3 mL of chloroform, vortexed for 1 min and subjected to 30 min of stirring in a rotary shaker. One milliliter of a KCl solution (0.2 M) was added to promote phase separation; after that, the falcon tube was centrifuged for 15 min at 3154× *g*. The organic phase (1 mL) was placed in a glass vial and evaporated under a gentle nitrogen stream. The lipid fraction was quantified by weight and expressed as g/100 g FW.

#### 2.4.4. Tocopherols Content

The homogenized sample (5 g) was weighed in a 50 mL falcon tube with 10 mL of heptane, vortexed for 1 min and stirred in a rotary shaker for 30 min. The tube was centrifuged for 15 min at 3154× *g*. The determination of the tocopherols was carried out by HPLC 1100 (Agilent Technologies, Milan, Italy) coupled with FLD detector. The column was a Licrosphere 100 (diol—5 µm Merck, Milan, Italy). The chromatographic separation was obtained under isocratic conditions using as eluent a mixture of heptane/TBME (95:5 *v*/*v*) at a flow rate of 0.3 mL/min. The injection volume was 20 µL and the total duration of the analysis was 45 min. Analysis was performed at 290 nm excitation and 330 nm emission. Quantitative determination was carried out by correlating the peak area with the concentration of the standard. The results were expressed in mg/kg of α-tocopherol FW.

#### 2.4.5. HPLC Analysis of Polyphenols

An amount of 5 g of homogenized samples were weighed in a 50 mL falcon tube plus 10 mL of a solution methanol/water (80:20, *v*/*v*). The tube was vortexed for 1 min and stirred in a rotary shaker for 15 min. After that, 2 mL of hexane was added to the sample and the extraction in the rotary shaker was repeated for 10 min before the centrifugation step (10 min at 3154× *g*). A volume of 1 mL of methanolic extract was transferred to a glass vial and analyzed by HPLC Agilent 1100 coupled with a diode array detector (DAD). The column was a Phenomenex C18 (5 μm, 150 mm × 4.6 mm) (Phenomenex, Castel Maggiore, Bologna, Italy), the flow rate was 0.3 mL/min, and the injection volume was set at 20 µL. The eluent mixture was composed by a solution of H_3_PO_4_ (0.22 M) (A) and a solution of methanol/acetonitrile (50:50, *v*/*v*) (B). The elution conditions were the following: T = 0 A 96%, B 4%; T = 40 A 50%, B 50%; T = 45 A 40%, B 60%; T = 60 A 0%, B 100%; T = 70 A 0%, B 100%; T = 71 A 96%, B 4%; T = 81 A 96%, B 4%; post time 5 min. Analyses were carried out at 280 nm for oleuropein, hydroxytyrosol and tyrosol, 313 nm for hydroxycinnamic acid derivatives, and 360 nm for flavanols. The polyphenols were identified by comparison with analytical standards, retention times and UV–Vis spectra. All data were reported as mg/kg FW and performed in triplicate.

#### 2.4.6. Total Phenolic Content

A volume of 200 µL of methanol/water extract were placed in a 10 mL volumetric flask with 500 µL of Folin–Ciocalteu reagent and 1 mL of a sodium carbonate solution (20% *w*/*v*) and stirred for 5 min at room temperature. Milli-Q water was added till a final volume of 10 mL. The samples were incubated for 80 min at ambient temperature and finally centrifuged at 3154× *g* for 10 min. The obtained solution was transferred into a 1 cm quartz cuvette for spectrophotometric analysis. The quantification of the phenolic fraction was carried out by using a Varian Cary50 spectrophotometer (Varian, Palo Alto, CA, USA) at 725 nm. The quantification was carried out correlating the absorbance (Abs) to the concentration of gallic acid as the reference standard. The results were expressed as equivalents of gallic acid (mg/kg FW).

#### 2.4.7. Chloride Content

Chloride content was determined according to the Volhard method. An amount of 1 g of homogenized sample was digested in a 250 mL conical flask with 20 mL of HNO_3_ 65% plus 20 mL of AgNO_3_ 0.1 N at 60 °C under constant stirring. Digestion was considered finished when no traces of the sample were found and a white precipitate of AgCl settled on the bottom of the flask. A volume of 50 mL of Milli-Q water and 2 mL of ferric alum were added to the solution as an indicator. The sample was back titrated by using ammonium thiocyanate 0.1 N. The concentration of chlorides was expressed as g/100 g of NaCl.

### 2.5. Starter Culture Origin and Preparation

The freeze-dried starter culture (SIE) used in this study was previously used and described, [7,18]. It is a multi-strain culture of *Lactiplantibacillus pentosus*, tested for its capacity to withstand high salinity levels, to grow at low temperatures, and the ability to degrade oleuropein. SIE culture was grown in MRS broth at 30 °C in anaerobiosis. Following overnight incubation, was centrifuged (Centrifuge SL40R, Thermo Fisher Scientific, Lagenselbold, Germany) at 4500 rpm for 15 min at 4 °C. After discarding the supernatant, the resulting pellet was washed with 200 mL of sterile saline solution (0.85% *w*/*v* NaCl) to remove residual growth medium. The washed pellet was then resuspended in a cryoprotectant solution composed of 5% gelatin, 5% Na-citrate, 5% monosodiumglutamate, and 10% sucrose, at pH 7. Then, the culture was frozen at −80 °C and later subjected to freeze-drying using a CoolSafe 55-4 Pro (Labogene, Lillerød, Denmark). The resulting freeze-dried culture was divided into equal aliquots (13 g of lyophilized culture for each barrel) to prepare the final starter culture for fermentation trials. At the time of inoculation, each aliquot was dispersed in 100 mL of the same brine used for processing, allowed to dissolve through gentle agitation, then mixed to the barrels’ content, to reach an approximate final cell concentration of 5 Log CFU/mL.

### 2.6. Microbial Analysis

Samples constituted by 130 g of olives and 90 mL of fermentation brine were homogenized for 10 min with a BagMixer paddle blender (Interscience Corporation, Saint Nom, France), dispensed in decimal serial dilutions in saline solution (0.89% *w*/*v* NaCl), then plated on agar media specific for different microbial groups. Mesophilic lactobacilli were enumerated on FH agar, incubated at 37 °C for 72 h in anaerobiosis; molds and yeasts on MEA agar (Microbiol, Uta, Cagliari, Italy) supplemented with 0.01% of chloramphenicol (Sigma-Aldrich), incubated at 25 °C for 3–5 d in aerobiosis; enterobacteria on VRBGA (Microbiol), incubated at 37 °C for 18–24 h in aerobiosis. Microbial counts were performed in duplicate and microbial concentration was expressed as average Log CFU/mL. Analyses lasted up to 210 days after inoculation.

### 2.7. Texture Analyses

Texture profile analyses (TPA) were carried out with a TA-XT Plus texture analyzer (Stable Microsystems, Surrey, UK) with a plugged 30 kg load cell, coupled with the Exponent software (ver. 6.1.3.0) for acquisition and processing. Analyses were carried out on 30 fruits for each replicate, for a total of 90 fruits for each experimental condition. Olives were put on the heavy-duty platform and compressed along the longitudinal side by 15% of their thickness with the P/40 aluminum cylinder. Test speed was set at 1 mm/s, time between compressions was 2 s, and trigger force was set at 0.05 N. The TPA parameters computed were hardness, adhesiveness, cohesiveness, gumminess, chewiness and springiness, according to Szczesniak and Friedman et al. [19,20] (Figure S1).

### 2.8. Consumer Testing

An acceptability test was performed [21]. The test was carried out by 62 consumers, 26 women and 28 men, 32 to 63 years old, recruited based on interest and willingness. They were regular consumers (one time a month, at least) of table olives, not trained in sensory analysis. In total, 3 olives in brine were supplied to each consumer, who were asked to give a judgment for acceptability by scoring samples using a nine-point structured hedonic scale ranging from 1 (extremely disliked it) to 9 (extremely liked it). A sample was considered acceptable when it scored above 5 (neither like nor dislike). Unsalted crackers and water were supplied to rinse the mouth between tested samples.

### 2.9. Statistical Analyses

A 2 × 2 full factorial design (inoculum, temperature) and 3 replicates (barrels) were used, with three repetitions (analyses). The GLM ANOVA (General Linear Model) procedure, with interaction between factors, was used to discriminate between means, and Tukey’s HSD (honestly significant difference) as post hoc test, at *p* ≤ 0.05. The same procedure was performed to evaluate significant (*p* ≤ 0.05) differences of microbial concentrations, texture parameters and sensory scores, among the four treatments. The software XLStats (Premium 2023.1.4) was used to perform statistical analyses.

## 3. Results and Discussion

### 3.1. Chemical–Physical Characteristics

**Figure 1:**
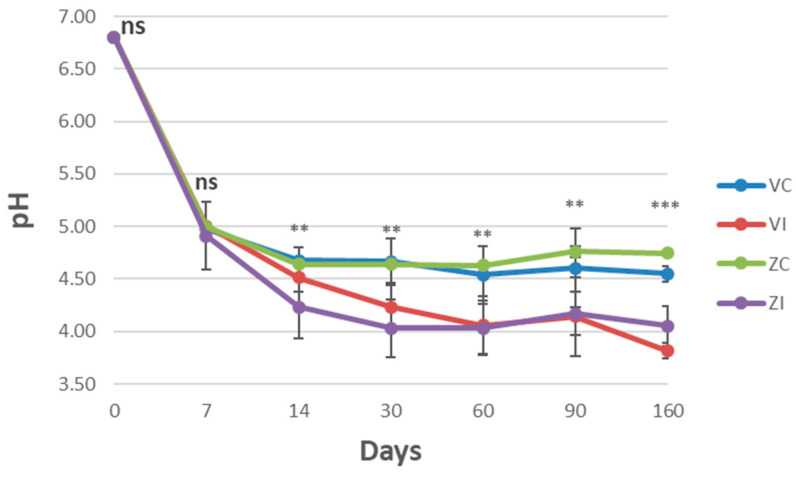
pH values during the fermentation (mean ± standard deviation). Significance of Tukey’s test after ANOVA (*p* ≤ 0.05): ns, not significant; *, significant for temperature; **, significant for starter; ***, significant for temperature × starter.

pH is a key parameter for microbial growth and compound synthesis, ensuring proper fermentation and hindering the proliferation of pathogenic and spoilage microorganisms. Different species of microorganisms have different pH requirements. The analysis of the pH on brine during table olive processing showed a different behavior between uninoculated (control) and inoculated samples (Figure 1). The pH trend was very similar among the inoculated samples (VI, ZI), regardless of storage temperature, with values ranging at the end of the process from 3.81 to 4.05. It was well below the safety pH for this type of preparation (4.2) already after 30 days of processing ranging from 4.03 to 4.23, ensuring a rapid disappearance of the spoilage forms. On the contrary, the pH of the uninoculated samples (VC, ZC) showed at 160 d values ranging from 4.55 to 4.74, and above 4.5 during all experiment, this fact was probably due to the stunted growth of lactobacilli in the fermenting medium (Figure 1).

These results confirm previous observations made with the same starter culture and olive variety under similar fermentation conditions at the laboratory scale [7]. In those earlier experiments, we observed a rapid decline in pH in inoculated samples, reaching values <4 within 12 days, whereas NF samples only reached pH 4.3 after 45 days. In the present study, the pH decrease was slower; however, after 30 days, the same pH values were achieved as in the laboratory tests. This difference can be attributed to greater variability in salt concentration, temperature and microbial distribution in larger tanks, compared to the laboratory-scale fermentations, resulting in increased variability in the outcomes. The salt concentration of the brine was kept constant during the process in all experiments, with values ranging from 6 to 7.5% by adding concentrated brine, when necessary. This concentration prevents the onset of abnormal fermentations, since 6% NaCl concentration corresponds to an aw of 0.964, which is below the limit of *Clostridium* type E and Enterobacteriaceae tolerance, and at the same time preserves the antioxidant capacity compared to lower salt concentrations (4% *w*/*w*) [22]. The solubility of oxygen species in saline solutions is dependent on the amount of dissolved electrolyte and the temperature. It increases with decreasing salinity and increasing temperature. Therefore, low salt concentrations can accelerate the polyphenols oxidation processes [23]. Martín-Vertedor et al. (2021) explored the impact of the processing temperature of Spanish-style table olives ‘Manzanilla de Sevilla’ and ‘Manzanilla Cacereña’ olives, concluding that maintaining brine at 20–24 °C during a 3-month period led to optimum firmness, better color indices, and greater free acidity and LAB populations, compared to an unheated control, speeding the fermentation process at the same time [14]. Similar conclusions were achieved by Cabrera-Banegil et.al (2018) on Manzanilla Cacereña, who reported improvements of the final product quality by maintaining constant temperatures around 24 °C, decreasing table olives alterations, and increasing flavor development [24]. The results of the present study evidenced that the use of native microbial starter in the fermentation of table olives makes the heating of processing rooms unnecessary to achieve the safe technological pH, especially as in our case in the presence of low natural inoculum of lactobacilli, which are present in a small number on the fruit surface.

### 3.2. Olives Composition

The amount of moisture was temperature and inoculum dependent. Olive samples kept at controlled temperature showed lower moisture values over time (Table 1), compared to olives transformed at ambient temperature, whereas those with the inoculum showed higher moisture content with respect to the olives without starters, and these values were temperature dependent, in the later stages of fermentation. At the final collecting time, the samples fermented at ambient temperature showed higher moisture content than those with T °C controlled with and without the starter. In addition, after 160 d, the samples with the starter had higher moisture content than those without. Olives in brine undergo osmotic processes of dehydration, as they are submerged in a hypertonic medium, which is accelerated by temperature [16]. Analysis on total lipids showed values not influenced by temperature or starter addition except at the final collection time, which showed higher values in the sample with controlled temperature ranging from 11.3 ± 11.2% (mean ± RSD%) (VI 30 d) to 18.8 ± 4.6% (ZC 15 d). Tocopherols are important food antioxidants produced endogenously in olive; among them, the most represented in olives is α -tocopherol, accounting for almost 85% of total tocopherol content. Analysis data showed a similar trend for ZC, ZI, and VC, with the concentration of α-tocopherol steadily growing up to 60 d and decreasing until the end of fermentation to a final concentration slightly higher than the starting fresh material (10.4 ± 3.3 mg/kg). VI samples showed a constant level in the first 30 d, then increased to a maximum at 45 d, dropping below the initial values after 160 d (Table 1).

Due to its lipophilic nature, alpha tocopherol is not subject to migratory phenomena towards the brine; its levels could be influenced by the cultivar, the ripening stage and the processing technology [25]. Few authors have evaluated the evolution of tocopherols in table olives during fermentation. Hassapidou et al. (1994) compared different cultivars by analyzing olives after the debittering process and after fermentation in brine, obtaining almost comparable results [26]. Conversely, according to Sakouhi et al. (2008), a slight decrease during the fermentation process occurred in three different table olives evaluated during the green, cherry, and black stages [25]. The levels of NaCl in the olives increased in the olives till 90 d to around 2.5, then decreased. Driven by a slow diffusion process from the brine to the pulp of the drupes, the salt concentration increased throughout the fermentation from 0.3 in the fresh olives, reaching, in all experiments, the maximum concentration after 90 days (ranging from 2.2 ± 11.4% in ZC to 2.6 ± 5.0% in VC). However, at 160 d the concentration decreased to values between 1.0 and 1.8, giving the final product a relatively low salt content. NaCl, primarily acts as a preservative, lowers water activity (aw), inhibits the growth of undesirable microorganisms, increases the ionic strength of the brine, thereby reducing oxygen solubility in water, promotes competitive and selective microbiological growth, ensures microbial safety during storage, and enhances the sensory properties of the final product [27]. However, excess dietary salt is considered the most critical factor responsible for essential hypertension. The World Health Organization (OMS) recommends levels of 2 g/d of sodium intake, corresponding to approximately 5 g of table salt.

### 3.3. Polyphenolic Profile

The concentration and polyphenolic profile of olives are strongly influenced by cultivar, agronomical practices, bioclimate, etc. Othman et al. (2009) reported an important decrease in total polyphenolic concentration after both spontaneous fermentation in brine and after the inoculation of *L. plantarum* [28]. Indeed, water-soluble compounds, such as polyphenols, vitamins, and ions, are transported out of olives by the osmotic difference between the olive and brine. Nevertheless, Sherahi et al. (2018) observed an opposite behavior after treatment with the same species, since the total polyphenols content grew during the entire fermentation process [29]. This fact may be related to the ability of *Lactiplantibacillus* spp. (the late “*Lactobacillus*” genus) to “trap” the oxygen presents in the solution, responsible for the auto-oxidation of polyphenols [30]. In addition, the enzymes released by bacteria during fermentation drive the degradation of complex polyphenols to small molecules, leading to an increase in total phenolic content. D’Antuono et al. (2018) reported that the presence of a thin layer surrounding the pulp could influence the migration of polyphenols into the brine [31]. We observed fluctuating total polyphenol values during the experiment, suggesting a probable balance between the mechanisms proposed by the other authors. Samples maintained at controlled temperature showed higher amounts of total polyphenols at the end of fermentation, with values ranging from 3223 ± 12.3 mg/kg (VC—7 days) to 3637 ± 9.9 mg/kg (VI—7 days). In addition, VI showed the highest values of the whole trial. This fact can be explained by the development of a biofilm in inoculated olives acting as a barrier for the phenolic compound’s diffusion [28]. Fresh untreated olives accounted for 3374.4 ± 1.2 mg/kg. Thus, the fermentation process with the starters seems to preserve the entire phenolic fraction, ensuring a healthy and quality final product. Data from HPLC analysis, obtained at the end of fermentation, followed a similar trend but showing mean values lower (Table 2).

The Folin–Ciocalteu method leads to an overestimation of the polyphenol concentration due to the detection of other analytes, resulting in slightly higher values than those quantified by HPLC. ANOVA showed a significant interaction between temperature and microbial starter at the end of the trial. The primary process involving polyphenols is the degradation of oleuropein to form hydroxytirosol and elenolic acid by β-glycosidases and esterases enzymes’ activities. Oleuropein is responsible for the bitter taste of unripe olives and is reported to be one of the most concentrated polyphenols in fresh olives. The inoculation with microbial starter is the only significant determinant for the differences in final hydroxytyrosol content, which is the most abundant phenolic compound detected at the end of the process (Table 2). The inoculated samples showed the highest increase in hydroxytyrosol concentration (2.2 times), confirming data obtained for pH; an appropriate choice of the native microbial strain can allow the olives to be de-bittered avoiding unnecessary costs for maintaining controlled temperature. Several authors reported an increased concentration of hydroxytyrosol in olives processed with oleuropein-lytic bacteria [32,33,34]. Qualitative analysis of polyphenols performed by HPLC-DAD highlighted the presence of 15 main compounds. After 160 days of fermentation, the most concentrated polyphenols were hydroxytyrosol, luteolin, verbascoside and some of its derivatives (Table 2). Other compounds found in appreciable concentrations were apigenin, rutin and a derivative of hydroxycinnamic acid. Coumaric acid, detected and quantified in VC and VI samples, was not detected in the samples processed at ambient temperature. Apigenin was the less abundant analyte with values accounting for approximately 2 mg/kg (Table 2). The concentrations of hydroxytyrosol, tyrosol, verbascoside and its derivative, apigenin and luteolin increased at the end of the fermentation. Tyrosol showed final concentrations from 2.7 (VI) to 3.8 (ZC) times higher than those of olives sampled after 7 days. The increase in luteolin concentration was related to the contemporary decrease in luteolin 3-O-glucoside, probably due to the loss of the glucoside group, whereas oleuropein was metabolized, leading to an increase in the concentration of hydroxytyrosol. The inoculum included strains with a marked esterase activity [18]. Rutin underwent a strong decrease in concentration due to the loss of the disaccharide rutinose group; however, no trace of quercetin derivative was found.

### 3.4. Microbial Analyses

**Figure 2:**
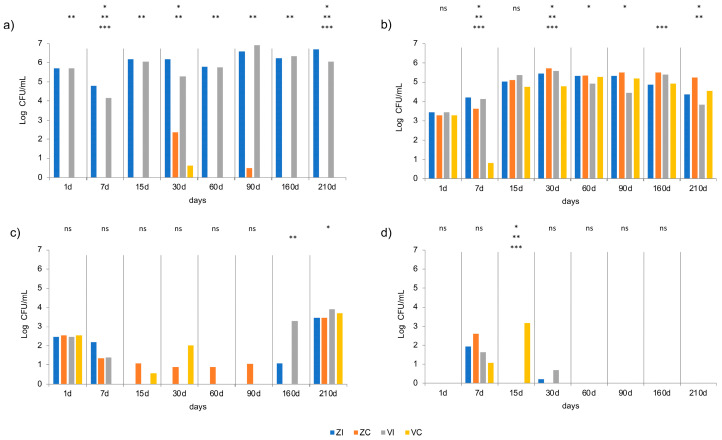
Microbial counts (Log CFU/mL) of mesophilic lactobacilli (**a**), yeasts (**b**), molds (**c**), and Enterobacteriaceae (**d**). Blue: ZI; orange: ZC; grey: VI; yellow: VC. For each time of analysis, significance of Tukey’s test after ANOVA (*p* ≤ 0.05) is reported above histograms: ns, not significant; *, significant for temperature **, significant for starter; ***, significant for temperature × starter.

Microbial population dynamics were monitored throughout the table olive fermentation process, comparing conditions with starter culture inoculation (I) in brines at 5 Log CFU/mL, and without inoculation (C), as a control. LAB were significantly (*p* ≤ 0.05) higher in inoculated brines (ZI and VI) compared to non-inoculated controls (ZC and VC) throughout the fermentation process (Figure 2a), confirming the importance of inoculation in promoting a faster and more controlled fermentation, as previously reported by [18]. These findings also align with earlier studies highlighting how the use of starter cultures can enhance the stability and safety of the fermentation process, reducing variability across different production facilities [7,17,35]. In non-inoculated samples, LAB populations developed more slowly, with significantly lower counts observed between 7 and 15 days. The absence of initial inoculation leads to unfavorable microbial competition, limiting LAB growth and potentially allowing for the proliferation of undesirable microorganisms. Indeed, in ZC and VC, LAB counts remained lower even after 30 days and showed a significant decrease at certain time points. Conversely, in inoculated brines, the bacterial population remained high and stable until the end of fermentation at 120 days, thus indicating effective control over the microbiota, with direct implications for the quality of the final product. Yeast populations exhibited a more variable trend across conditions. At the beginning of fermentation, yeast counts were similar in inoculated and non-inoculated batches after 7 days, yeasts in VC were significantly (*p* ≤ 0.05) lower than in VI and the other batches, ZI and ZC (Figure 2b). Moreover, non-inoculated brines showed higher yeast populations, though not always significant, likely due to reduced competition from LAB. ZC samples exhibited a more pronounced increase in yeast counts compared to VC, at 210 d, suggesting facility-dependent variability in indigenous microbiota composition. Similar findings regarding competition between LAB and yeasts influencing the microbial balance during fermentation were reported by Vega Leal-Sanchez et al. [36]. Molds were detected only in the early stages of fermentation, with very low counts and a rapid decline within the first month (Figure 2c), indicating a more rapid decline in inoculated samples compared to non-inoculated ones.

Inoculated batches (ZI and VI) showed a more rapid mold reduction, suggesting a potential competitive effect from the starter culture, contributing to a safer fermentation process. In contrast, non-inoculated samples, particularly in ZC, exhibited a presence of molds, still detectable at 30 days, albeit at low concentrations, that may indicate a higher risk of product spoilage. At 160 and 210 d, mold concentration increased, first in the inoculated vats ZI and VI, and then in all vats, reaching 4 Log UFC/mL. Enterobacteriaceae were detected during the initial week of fermentation, but their counts decreased over time (Figure 2d). In inoculated brines (ZI and VI), their reduction was significantly faster, with a marked reduction observed by days 7–15 and complete absence after the first month of fermentation. In contrast, non-inoculated samples (ZC and VC) showed slightly greater persistence of Enterobacteriaceae, particularly in VC, where their counts were higher than in ZC. These findings suggest that starter culture inoculation facilitated a more rapid pH drop, likely contributing to the effective inhibition of Enterobacteriaceae populations and are consistent with previous studies demonstrating that lower pH levels can limit the growth of Gram-negative bacteria during fermentation [37]. The general linear model analysis (LGLM) always evidenced a significant (*p* ≤ 0.05) effect of the inoculum for mesophilic lactobacilli, and the temperature × inoculum interaction was significant at 7 and 210 days (Figure 2). For yeasts, a general effect of temperature, inoculum and the interaction temperature × inoculum, at 7, 30 and 160 days, was observed. The inoculation of brines with starter culture had a significant impact on the microbial composition of table olive fermentation, promoting the rapid growth of LAB and accelerating the reduction in Enterobacteriaceae and molds, improving product quality and safety. The use of starter cultures contributes to a more controlled and safer fermentation process, reducing variability between different facilities, reducing pH faster, enhancing microbial safety, and improving sensory properties compared to natural fermentation [10,11]. Moreover, starter cultures, particularly former *Lactobacillus* genus strains, have been extensively studied for their potential as starter cultures, with some strains showing probiotic characteristics [2,6]. These cultures can help control the fermentation process, reduce spoilage risks, and potentially create functional foods, also aiming to enhance product quality, safety, and nutritional value while meeting consumer demands for natural and healthy foods [8].

### 3.5. Texture Analyses

The TPA test highlighted the influence of temperature, and temperature*inoculum interactions on the differences in Texture profile. The main differences were found in hardness, gumminess and chewiness, which resulted significantly higher in samples processed at ambient temperature (Table 3).

The samples processed at ambient temperature showed a significantly higher resistance to deformation, as shown by the hardness, gumminess and chewiness parameter magnitudes. As previously reported, changes in texture during natural fermentation of olives can be ascribed to hydrolysis of cell wall pectic polysaccharides, which results in loss of structural coherence of olive tissues, consistent with what has been observed by Servili et al., 2008, with SEM techniques [38,39]. Microorganisms can play a significant role in the textural changes in table olives. Among frequently isolated species, strains of *Saccharomyces cerevisiae* and *Wickerhamomyces anomalus* have been thought to be responsible for producing high amounts of CO_2_, leading to gas-pocket defects. Additionally, *Debaryomyces hansenii*, *Wickerhamomyces anomalus*, and certain species of the *Rhodotorula* genus have shown the ability to degrade fruit cell wall polysaccharides or to produce extracellular enzymes such as proteases, xylanases and pectinases causing olives softening [40,41]. Our data suggest that all other experimental conditions being equal, these phenomena are temperature dependent, and could affect negatively the textural properties, since olives that are softer, with an incoherent structure, are less appreciated by consumers.

### 3.6. Consumer Testing

Sensory quantitative descriptive analysis (QDA) using trained panels is recurrent in the literature for table olive quality evaluation [16,35,42,43]. However, only limited reports can be found on consumer testing results reporting the acceptability of samples inoculated with starter cultures. The results of the consumer test showed that all samples resulted more than acceptable (average hedonic judgment >5; VI: 5,52; VC: 5,76; ZI: 5,80; ZC: 6,59), although ZC resulted significantly more accepted (Table 4).

Statistical analyses showed that both starter and temperature have a significant influence on the acceptability. Although no descriptive tests were run, consumers were allowed to comment on the samples tested, and recurring comments were that inoculated olives were often “sparkling”, that is, the feeling associated with drinking carbonated beverages, and “more acidic”, in terms of detection of the acid taste in the mouth. This is related to the higher CO_2_ production in inoculated batches, and higher lactic acid production, respectively. Moreover, samples (VC, VI) processed at controlled temperature were often found as “softer”, confirming the results of TPA analyses. This could be related to a higher extent of cell wall damage occurring because of higher processing temperatures. Also, the higher rate of fermentation leads to a temporary accumulation of CO_2_ inside the olives, causing a degree of swelling and subsequent collapse of the tissues. These phenomena must be considered for the “fine tuning” of microbial starter composition and processing temperatures adopted, in relation to the sensory quality of the final product. From our results, when microbial starters are used, it could be advisable not to heat the processing chambers in the first stages of the fermentation (2 weeks), since a fast and “tumultuous” fermentation has a negative impact on texture. Moreover, the massive amount of CO_2_ produced in the first stages must be allowed to exit by partially closing the fermentation vessels.

## 4. Conclusions

This research provides compelling evidence for the benefits of incorporating microbial starters in table olive fermentation industrial practices. Modernizing olive fermentation processing by carefully selecting microbial strains and optimizing their interactions with other processing parameters, such as temperature conditions, is crucial for achieving desired fermentation outcomes on an industrial scale. This study highlights the role of microbial starters in promoting faster fermentation, even at ambient temperatures. This advancement can significantly reduce production costs by eliminating the need for energy-intensive heating systems and by mitigating the risk of fermentation failure caused by insufficient LAB populations, particularly during colder seasons. Autochthonous starter cultures demonstrated robust growth even under suboptimal conditions, offering a cost-effective solution for producers without the infrastructure to heat fermentation rooms or brine, and the subsequent reduction in energy consumption provides significant economic advantages. Overall, this approach leads to improved production efficiency, enhanced process reliability, and greater product consistency, contributing to more efficient and sustainable production practices. However, the impact of starter cultures on sensory acceptability, in particular texture characteristics, must be carefully evaluated, since their use could influence key sensory attributes. Therefore, industrial producers are encouraged to adopt effective microbial starters to improve process control, enhance food safety, and reduce manufacturing costs, while also considering potential effects on product quality.

## Figures and Tables

**Table 1:** Composition of olive samples through the fermentation process. Values are the means ± RSD%. T, temperature; I: inoculum; T × I: factors interaction. ns: not significant; * significant for *p* ≤ 0.05.

Factors	Samples	Moisture	Total Lipids	Tocopherols	NaCl
		(g/100 g)	(g/100 g)	(mg/kg)	(*w*/*w*)
	ZC 7 d	65.7 ± 1.5	16.5 ± 6.4	9.6 ± 3.9	1.1 ± 3.4
	ZI 7 d	65.1 ± 1.9	13.5 ± 17.1	11.2 ± 1.5	0.9 ± 14.4
	VC 7 d	64.4 ± 0.65	13.7 ± 8.7	11.7 ± 9.1	0.9 ± 7.3
	VI 7 d	65.8 ± 1.0	16.0 ± 6.2	18.1 ± 13.0	1.0 ± 12.6
Temperature (T)		ns	ns	*	
Inoculum (I)		ns	ns	*	
T × I		ns	ns	*	
	ZC 14 d	64.1 ± 0.4	18.8 ± 4.6	13.7 ± 9.5	1.2 ± 8.9
	ZI 14 d	64.4 ± 2.9	17.0 ± 5.1	17.6 ± 8.6	1.0 ± 9.9
	VC 14 d	64.8 ± 0.8	17.6 ± 5.4	14.8 ± 4.2	1.2 ± 9.8
	VI 14 d	65.2 ± 1.1	13.5 ± 17.1	17.7 ± 1.1	1.2 ± 11.0
Temperature (T)		ns	ns	ns	
Inoculum (I)		ns	*	*	
T × I		ns	*	ns	
	ZC 30 d	64.1 ± 2.0	14.5 ± 5.9	21.6 ± 4.7	1.8 ± 17.2
	ZI 30 d	64.7 ± 2.7	14.9 ± 9.0	20.9 ± 2.5	1.3 ± 15.7
	VC 30 d	63.0 ± 0.3	15.4 ± 7.9	21.7 ± 3.9	1.3 ± 12.1
	VI 30 d	64.7 ± 1.7	11.3 ± 11.2	16.5 ± 11.2	1.1 ± 16.0
Temperature (T)		ns	ns	ns	
Inoculum (I)		ns	ns	ns	
T × I		ns	ns	ns	
	ZC 45 d	64.7 ± 1.3	16.2 ± 7.0	38.9 ± 9.3	1.8 ± 11.9
	ZI 45 d	64.4 ± 1.3	16.1 ± 3.8	30.5 ± 4.1	1.7 ± 16.0
	VC 45 d	65.6 ± 3.1	14.6 ± 9.8	41.4 ± 0.7	1.0 ± 5.7
	VI 45 d	63.9 ± 2.1	14.9 ± 8.8	32.1 ± 18.3	1.3 ± 14.4
Temperature (T)		ns	ns	ns	
Inoculum (I)		ns	ns	*	
T × I		ns	ns	ns	
	ZC 60 d	64.8 ± 1.0	16.9 ± 16.4	39.0 ± 4.4	1.9 ± 10.8
	ZI 60 d	65.7 ± 0.6	15.3 ± 10.4	37.3 ± 5.8	2.2 ± 7.7
	VC 60 d	63.9 ± 0.8	16.6 ± 9.0	40.7 ± 15.7	1.8 ± 10.4
	VI 60 d	62.9 ± 1.6	17.2 ± 7.5	20.5 ± 1.3	1.4 ± 9.0
Temperature (T)		*	ns	*	
Inoculum (I)		ns	ns	*	
T × I		*	ns	*	
	ZC 90 d	65.3 ± 1.6	12.0 ± 4.0	16.2 ± 8.5	2.2 ± 11.4
	ZI 90 d	67.4 ± 2.1	12.0 ± 8.3	15.8 ± 4.6	2.4 ± 12.8
	VC 90 d	63.1 ± 0.7	13.2 ± 10.4	15.3 ± 7.0	2.6 ± 5.0
	VI 90 d	63.2 ± 2.0	13.4 ± 0.8	16.2 ± 9.4	2.4 ± 8.1
Temperature (T)		*	ns	ns	
Inoculum (I)		ns	ns	ns	
T × I		ns	ns	ns	
	ZC 160 d	67.0 ± 2.9	14.6 ± 5.7	12.8 ± 5.6	1.3 ± 4.9
	ZI 160 d	67.9 ± 2.2	13.5 ± 2.0	14.4 ± 7.5	1.8 ± 3.2
	VC 160 d	62.6 ± 1.0	16.2 ± 9.5	15.8 ± 9.6	1.6 ± 5.5
	VI 160 d	64.8 ± 1.4	17.3 ± 1.8	12.8 ± 2.1	1.0 ± 3.4
Temperature (T)		*	*	ns	
Inoculum (I)		ns	ns	ns	
T × I		ns	ns	*	

**Table 2:** Phenolic compounds identified in olive samples through the fermentation process. Values are the means ± RSD%. T, temperature; I: inoculum; T × I: factors interaction. T, temperature; I: inoculum; T × I: factors interaction. ns: not significant; nd: not detected; * significant for *p* ≤ 0.05 (Tukey’s test). ^a^ quantified as verbascoside; ^b^ quantified as coumaric acid; ^c^ quantified as luteolin glucoside.

Sample	ZC		ZI		VC		VI		Factors (7 d)			Factors (160 d)		
Sampling Time (d)	7	160	7	160	7	160	7	160						
Compounds	mg/kg	mg/kg	mg/kg	mg/kg	mg/kg	mg/kg	mg/kg	mg/kg	T	I	T × I	T	I	T × I
Hydroxy-tyrosol	238.3 ± 4.3	480.7 ± 7.3	322.5 ± 5.1	719.2 ± 25.3	435.1 ± 10.7	326.1 ± 2.8	337.8 ± 9.3	762.9 ± 9.0	*	ns	ns	ns	*	ns
Tyrosol	30.2 ± 10.3	114.8 ± 3.2	38.8 ± 14.9	113.1 ± 8.3	43.9 ± 3.4	143.3 ± 4.7	38.1 ± 7.7	105.5 ± 5.5	ns	ns	ns	*	*	*
Verbascoside der. ^a^	166.6 ± 7.9	390.1 ± 17.3	129.5 ± 5.6	297.3 ± 0.1	205.7 ± 6.9	451.5 ± 12.3	136.3 ± 1.7	293.3 ± 12.9	ns	ns	ns	ns	*	ns
Verbascoside der. ^a^	130.9 ± 6.5	269.2 ± 3.9	125.5 ± 17.5	193.2 ± 6.5	217.0 ± 11.5	411.4 ± 5.6	138.0 ± 7.4	215.4 ± 8.1	ns	ns	ns	*	*	ns
Verbascoside der. ^a^	311.5 ± 18.8	412.9 ± 3.2	349.3 ± 11.9	179.7 ± 10.9	588.3 ± 2.1	27.4 ± 2.2	445.4 ± 6.1	138.0 ± 5.7	*	ns	ns	ns	*	*
Verbascoside	256.4 ± 13.4	194.4 ± 25.9	293.0 ± 4.6	396.6 ± 18.7	nd	333.4 ± 6.8	434.8 ±	409.0	*	ns	ns	ns	ns	ns
Coumaric acid	nd	nd	nd	nd	104.1 ± 5.8	54.8 ± 8.3	118.2 ± 0.5	135.2 ± 35.6	*	ns	ns	*	*	*
Rutin	218.3 ± 6.3	85.2 ± 10.2	263.3 ± 12.8	32.3 ± 5.7	368.6 ± 18.4	nd	322.6 ± 5.6	nd	*	ns	ns	ns	*	ns
Lutelolin glucoside	511.4 ± 6.0	214.0 ± 17.6	563.2 ± 3.4	244.6 ± 31.0	733.6 ± 14.4	277.6 ± 17.1	604.1 ± 11.1	120.6 ± 2.4	*	ns	ns	ns	ns	ns
Oleuropein	210.2 ± 22.5	99.7 ± 16.3	386.9 ± 23.5	123.9 ± 12.5	525.8 ± 10.9	115.7 ± 4.6	445.0 ± 17.1	74.3 ± 14.0	*	ns	ns	ns	ns	ns
Verbascoside der. ^a^	105.2 ± 8.2	66.9 ± 7.7	108.4 ± 9.7	89.7 ± 7.6	222.3 ± 9.8	64.5 ± 2.1	210.6 ± 21.7	82.6 ± 19.3	*	ns	ns	ns	ns	ns
Sinapic acid der. ^b^	183.1 ± 2.6	160.0 ± 6.8	176.9 ± 9.3	115.8 ± 7.4	302.9 ± 6.9	150.2 ± 3.7	207.9 ± 6.5	147.0 ± 12.7	*	ns	ns	ns	*	ns
Luteolin ^c^	189.0 ± 5.5	342.5 ± 9.0	183.1 ± 1.9	339.9 ± 14.2	146.0 ± 6.6	296.2 ± 5.3	158.7 ± 25.7	304.9 ± 2.7	ns	ns	ns	ns	ns	ns
Apigenin	2.7 ± 2.2	4.4 ± 17.9	2.8 ± 4.6	4.3 ± 15.6	2.1 ± 8.0	3.6 ± 2.6	2.4 ± 13.8	3.6 ± 15.4	ns	ns	ns	*	ns	ns
**Total polyp. HPLC**	**2553 ± 7.6**	**2834 ± 1.7**	**3158 ± 10.9**	**2534 ± 15.3**	**4079 ± 14.4**	**2659 ± 8.1**	**3271 ± 3.4**	**2664 ± 20.6**	*	ns	*	*	ns	ns
**Total polyp. Folin**	**2906 ± 6.3**	**3138 ± 8.6**	**2350 ± 6.4**	**2112 ± 1.3**	**3223 ± 12.3**	**3458 ± 10.1**	**3637 ± 9.9**	**3622 ± 2.6**	*	ns	*	*	*	*

**Table 3:** TPA parameter. Values are the means ± RSD% (*n* = 30). T, temperature; I: inoculum; T × I: factors interaction. ns: not significant; * significant for *p* ≤ 0.05 (Tukey’s test).

TPA Parameter	VC	VI	ZC	ZI	T	I	T × I
Hardness (gr)	1651.2 ± 29.89	1771.56 ± 23.26	2290.84 ± 24.15	2102.44 ± 24.43	*	ns	*
Adhesiveness (g.s)	−1.23 ± 113.01	−2.42 ± 186.6	−3.15 ± 2.53	−2.77 ± 223.5	*	ns	ns
Springiness	0.74 ± 8.11	0.73 ± 10.96	0.72 ± 5.56	0.69 ± 11.59	*	*	ns
Cohesiveness	0.63 ± 6.35	0.61 ± 8.2	0.59 ± 5.08	0.58 ± 6.9	*	*	ns
Gumminess	1032.33 ± 30.05	1068.35 ± 22.83	1340 ± 17.14	1220.72 ± 23.78	*	ns	*
Chewiness	774.54 ± 33.78	786.28 ± 27.2	959.99 ± 21.74	844.74 ± 28.17	*	ns	*
Resilience	0.32 ± 12.5	0.32 ±12.5	0.31 ± 9.68	0.29 ± 13.79	*	ns	ns

**Table 4:** Average hedonic judgments from consumer testing. Values are the means ± RSD% (*n* = 62). T, temperature; I: inoculum; T × I: factors interaction. ns: not significant; * significant for *p* ≤ 0.05 (Tukey’s test).

Sample	Average Hedonic Judgments
VI	5.52 ± 32.56
VC	5.76 ± 25.66
ZI	5.80 ± 24.53
ZC	6.58 ± 20.39
Temperature	*
Inoculum	*
T × I	ns

## Data Availability

The original contributions presented in this study are included in this article, and further inquiries can be directed to the corresponding authors.

## Supplementary Materials

The following supporting information can be downloaded at: https://www.mdpi.com/article/10.3390/foods14132159/s1, Figure S1: TPA graph depicting the forces registered during the typical 2 compressions cycle and relative Texture parameter meaning and computing.
