# Supplementary material for: Efficacy of a Native Microbial Starter in Promoting Table Olive Fermentation: An Industrial-Scale Trial at Controlled and Ambient Temperature
Source: Foods. 2025 Jun 20;14(13):2159. doi: 10.3390/foods14132159 (PMC12248820; doi:10.3390/foods14132159)
Supplement: Supplementary file 1 [file foods-14-02159-s001.zip › foods-3679843-supplementary.pdf]

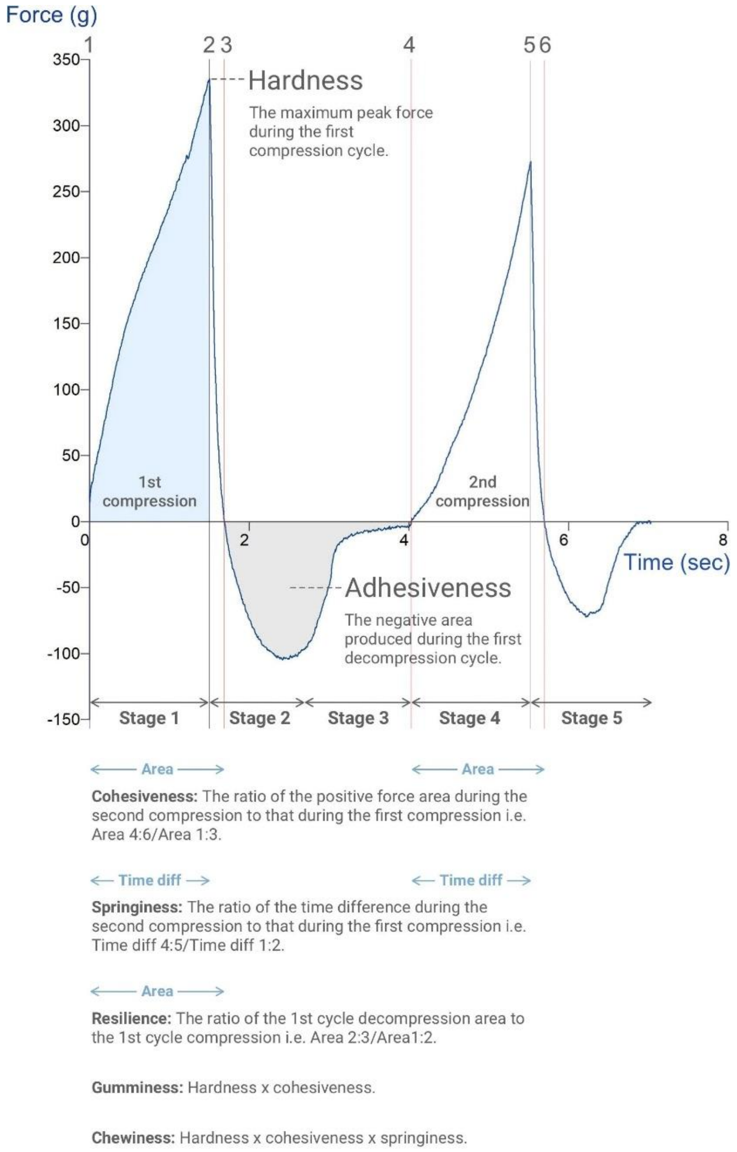

Figure S1. TPA graph depicting the forces registered during the typical 2 compressions cycle and relative Texture parameter meaning and computing.
